# The Care of Crippled Children

**Published:** 1920-11-20

**Authors:** 


THE PUBLIC WELL-BEING—(*continued*).

### The Care of Crippled Children.

### A LONDON CONFERENCE.

On Tuesday (writes a correspondent) I spent an interesting morning at the opening session of the joint London Conference of the Invalid Children's Aid Association and the Central Committee for the Care of Cripples. It was a businesslike as well as a sympathetic gathering, in which women predominated not only in point of numbers, but in the practical value of their contributions to the debate. Mr. H. A. L. Fisher, President of the Board of Education, was to have opened the proceedings with an address, and he would no doubt have received a very friendly hearing, for his Education Act does make provision for greatly increasing the efficiency of treating and caring for defective and crippled children. His place was taken by Mr. J. Herbert Lewis, who spoke with effect and understanding on the subject of the Conference.

In the discussion of the papers read by Mrs. Townsend and Miss Adler the blessed words "co-operation" and "co-ordination" were continually cropping up, but it is, after all, quite certain that until the voluntary agencies for dealing with physically defective children are properly linked up with State effort, the problem will never be solved. Miss Adler, who is a member of the London County Council, complained that in

**THE PUBLIC WELL-BEING**—(*continued*).

practice only a limited number of women attend the infant welfare centres. But under Section 19 of the Act of 1918 local authorities have a great power in their hands. They may aid nursery schools; and they are given full opportunities of caring systematically for the health, nourishment, and physical welfare of children, and providing medical treatment and education suited to their years and condition.

One of the greatest needs in London at the present time, in Miss Adler's opinion, is a residential hospital school just outside the Metropolitan area, with radiating clinics, which can be attended by children as out-patients. The serious difficulty of securing the attendance of helpless recumbent children at the out-patient departments of the hospitals is now generally realised. To employ an ambulance for the purpose, one speaker pointed out, costs £1 a day! The real problem in London and the great provincial cities is to provide continuous treatment over a long period. What the Conference sought to evolve, and what is the aim of the thousands of social workers interested in this question, is a well-thought-out scheme of medical treatment and education, by which these little children, instead of being allowed to develop into "useless parasites," may become healthy and efficient citizens able to enjoy life in wisely selected employment.

I hope to be able to return to the subject in more detail in a future issue.

